# How happy is healthy enough? Uncovering the happiness threshold for global non-communicable disease prevention

**DOI:** 10.3389/fmed.2025.1667645

**Published:** 2025-10-21

**Authors:** Iulia Cristina Iuga, Syeda Rabab Jafri, Horia Iuga

**Affiliations:** ^1^Department of Finance-Accounting, “1 Decembrie 1918” University of Alba Iulia, Alba Iulia, Romania; ^2^Center for Mathematical and Statistical Sciences, Lahore School of Economics, Lahore, Pakistan; ^3^Faculty of Medicine, “Iuliu Haţieganu” University of Medicine and Pharmacy, Cluj-Napoca, Romania

**Keywords:** happiness, non-communicable diseases, global health, life satisfaction, health policy

## Abstract

**Objectives:**

To determine whether the relationship between subjective wellbeing (happiness) and premature mortality from non-communicable diseases (NCDs) is non-linear and to identify the happiness level at which population health gains are maximized.

**Methods:**

A balanced panel of 123 countries (2006-2021) was analyzed. A Panel Smooth Transition Regression (PSTR) model examined the effect of national Life-Ladder scores on the 30-to-70-year NCD mortality rate, controlling for alcohol consumption, obesity prevalence, urbanization, PM2.5 exposure, health expenditure, GDP per capita and governance quality. The Life-Ladder index served as the transition variable, allowing coefficients to vary across two regimes separated by an estimated threshold. Dynamic feedback was explored with panel Vector-Autoregression (VAR) and impulse-response analysis.

**Results:**

The model identified a single threshold at 2.7 Life-Ladder points and rejected parameter constancy (LM-F = 8.85, *p* < 0.001). Above this level, each 1% rise in happiness decreased NCD mortality by 0.43% (*p* < 0.001); below it, the effect was nil. Obesity and alcohol consistently raised deaths, whereas urbanization turned from harmful to protective in the high-happiness regime. Health spending remained protective, and GDP per capita mattered only beyond the threshold. Complementary VAR and impulse-response analyses confirm a bidirectional negative linkage between happiness and NCD mortality and show that obesity, alcohol use and air pollution remain positive drivers of deaths. PVAR confirmed bidirectional Granger causality between happiness and NCD mortality and showed that a positive happiness shock elicits a sustained downward mortality response with no sign reversal.

**Conclusion:**

Subjective wellbeing (happiness) appears to function as a population health asset only once a minimum threshold of ~2.7 (on the 0–10 Life Ladder scale) is surpassed. Beyond this point, higher levels of happiness are associated with progressively lower NCD mortality, with no evidence within the observed range of adverse effects from “excessive” happiness. Public health strategies that elevate wellbeing above this tipping point-while concurrently addressing obesity, alcohol consumption, and environmental hazards-may initiate a reinforcing cycle of improved happiness and extended, healthier lifespans.

## 1 Introduction

Non-communicable diseases (NCDs) now account for approximately three-quarters of global mortality (1). While public-health initiatives focus primarily on combating tobacco use, poor nutrition, and inactivity, the salience of psychosocial wellbeing is increasing. The WHO defines health broadly, including mental and social flourishing, suggesting that happiness, in and of itself, might influence disease risk. The United Nations has similarly shaped its development agenda to include happiness and wellbeing (2), while some countries keep records of citizens' life satisfaction as a routine part of government (3). Consequently, an important research and policy question has emerged about whether subjective wellbeing protects against NCDs.

Evidence from economics and psychology has established a basis. The Easterlin Paradox revealed that once basic needs have been met, it is diminishing returns to additional happiness (4, 5). Layard (6) noted that modern societies need to pursue happiness, not just wealth. Indicators such as Cantril's life ladder show substantial cross-national differences in happiness, only somewhat attributable to income (3, 6).

A robust literature (7, 8) shows that greater subjective wellbeing —including life satisfaction, positive affect, and low stress—is associated with healthier behaviors or activity, enhanced immune function, reduced incidence of chronic disease. Biopsychosocial models specify a number of pathways for these effects: happier people are more likely to engage in health-promoting behaviors (e.g., more exercise and less smoking) as well as coped with stress more adaptively—this will lessen allostatic load (and inflammation) linked with NCD pathology (9). At the population level, cross-national studies find that higher average happiness (for a country) are linked to healthier health profiles (e.g., greater life expectancy; lower age specific mortality), even when income is controlled for (3, 10–12).

Recent studies conducted in Europe highlight the nuanced connections between wellbeing and health: Iuga et al. (13) found that certain macro-level indicators of wellbeing led to paradoxical outcomes in NCD outcomes. Iuga et al. (13) conducted a 27-country study where they found that higher GDP (a proxy for economic wellbeing) was associated with a higher prevalence of chronic diseases, and similarly, relative to GDP, higher health expenditures were associated with lower rates of diseases; they conclude that economic growth, without any accompanying preventative health implications, could produce inadvertent increases in risks to lifestyle-related NCDS. This so-called “economic prosperity and chronic disease paradox” (13) underscores that the relationship between wellbeing and health is not straightforward. It raises the question: *could* subjective *wellbeing (happiness) likewise have complex, non-linear effects on health outcomes?* In addition to psychosocial wellbeing, structural determinants such as economic inequality and corruption play a critical role in shaping public health outcomes, as evidenced in cross-national analyses (14).

Intuitively, one might expect that the happier people are, the healthier they become. To a point, this is supported by evidence—chronic stress and dissatisfaction are clearly detrimental, whereas moderate happiness and optimism are linked to favorable health markers (7, 9). However, emerging theoretical perspectives caution that *more* happiness is not always *better* in a linear fashion. It is possible to have “too much of a good thing” (15). Extremely high levels of happiness might lead to complacency or risk-taking behaviors that could undermine health—a notion backed by psychological studies showing that people who are *too* carefree or euphoric may neglect warnings or engage in unhealthy pleasures without due caution (16).

This current study examines if there is an optimal level of happiness that produces the greatest health benefits and reduces mortality from non-communicable diseases (NCD). Prior research suggests that very low wellbeing (i.e., depression, chronic stress) negatively affects health, while moderate increases in happiness can benefit health, though it is unclear that happiness continues to help health after certain increases or whether higher levels of happiness eventually stop helping—or even harm—population health. That uncertainty reflects a larger gap: most empirical work treats happiness as a linear predictor, rarely probing for non-linear dynamics or thresholds with large, cross-country panels. Previous empirical research has predominantly relied on linear specifications to examine the association between happiness and health outcomes [e.g., (17, 18)], implicitly if marginal changes in subjective wellbeing exert uniform effects across the entire wellbeing distribution. Such approaches risk overlooking potential threshold effects—critical points beyond which the health benefits of additional happiness may change in magnitude or even direction. Failure to identify these non-linearities could lead to mis-specified policy priorities, as interventions might be designed under the false premise that gains in happiness are equally beneficial for all populations, regardless of their baseline wellbeing. To close this gap, we analyse a global dataset for 2006–2021 using a Panel Smooth Transition Regression (PSTR) in which the Life Ladder index serves as the transition variable. This approach allows the coefficients on behavioral, demographic, environmental and economic-institutional covariates—and on happiness itself—to shift gradually between two regimes separated by an estimated threshold, thereby capturing potential diminishing or reversing returns. By locating the transition point and comparing the two regimes, we test whether national happiness continues to reduce NCD mortality indefinitely or whether its marginal health payoff eventually attenuates or reverses, offering policy-relevant insights into the balance between promoting happiness and improving public health.

In line with the above rationale, the main objective of this research is to test the non-linear relationship between subjective wellbeing (happiness) and health, specifically evaluating whether there is an optimal happiness level for minimizing NCD mortality. The analysis focuses on total NCD mortality as the key health outcome and uses the “happiness index” (based on life evaluation ladder scores) as the measure of subjective wellbeing. A Panel Smooth Transition Regression (PSTR) model is employed, controlling for a comprehensive set of covariates including behavioral factors (e.g., alcohol consumption, obesity prevalence), demographic and environmental factors (e.g., urbanization rate, air-pollution levels), and economic and institutional conditions (e.g., GDP per capita, healthcare expenditure, governance indicators). This flexible specification isolates the effect of happiness while allowing its impact—and that of the control variables—to shift smoothly between two regimes separated by an empirically estimated Life-Ladder threshold. To achieve the stated objective, the study is guided by two primary research questions: (1) *Up to what level does happiness have a significant beneficial impact on reducing NCD mortality?* (2) *At what point does the effect of increasing happiness become statistically insignificant or even adverse with respect to NCD-related health outcomes?* By addressing these questions, the study aims to pinpoint the “turning-point” level of happiness (if it exists)at which health benefits are maximized and beyond which further increments in happiness yield no additional gains. Identifying such a threshold moves beyond the simplistic assumption that more happiness is always better for health.

This investigation offers an original contribution to the interdisciplinary literature on wellbeing and public health in several ways. Firstly, it provides one of the first empirical assessments of a non-linear happiness–health relationship on a global scale. By examining data from over 120 countries across a 16-year period, this study extends prior research—often confined to single nations or short horizons (3, 19)—and tests whether the beneficial effects of happiness on reducing NCD mortality taper off at higher levels of happiness. Secondly, the study introduces a robust methodological framework that embeds subjective-wellbeing metrics within health-economics modeling. Specifically, it employs a Panel Smooth Transition Regression (PSTR) with extensive controls for behavioral, environmental and socio-economic factors, thereby strengthening causal inference and addressing confounders that earlier bivariate studies could not fully eliminate (7, 9). In doing so, it yields more nuanced evidence on how happiness independently correlates with health outcomes. Thirdly, the findings will fill a notable gap in both theory and practice by clarifying the threshold at which happiness maximizes health benefits.

This has direct policy implications: governments and international organizations increasingly see happiness or “gross national wellbeing” as policy goals, and knowing the “right” level of happiness to promote for health might be useful in directing health resources for the population. For example, if the findings indicate that very high happiness (beyond the identified optimal level) provides no additional survival advantage, then policy efforts might shift from improving happiness scores to also focussing on equity and qualiy of wellbeing in the population. Overall, by presenting new evidence on the complex role of happiness in shaping health trajectories, this study offers greater nuance in conceptualizing the happiness-health nexus, and provides evidence-based options re: public-health focus on improving population wellbeing and longevity.

## 2 Literature review

### 2.1 The happiness–NCD nexus: theoretical foundations and empirical evidence

One key mechanism linking happiness to NCD outcomes is health behavior. Higher subjective wellbeing is associated with positive health behavior (i.e., those who are happier exercise more often, eat healthy foods, do not smoke, and follow medical regimes that protect against chronic disease) (20). For instance, longitudinal studies indicate that having higher life satisfaction at baseline predicted maintaining a healthy behavior profile 9 years later in a prospective manner, building a virtuous cycle (while those lower on wellbeing areas more likely to be found engaging in harmful behaviors, indicating an interaction effect). People who were happier generally build deeper social support networks, which can foster healthy lifestyle choices and act as buffers during challenges when related to illness. These behavioral and social prospective factors may partially mediate the pathway to health, although are unlikely to be the only respective factors implicated, there may be non-behavioral or physiological paths (21).

Complementing these findings, Alnafrah and Belyaeva (22) uncover a non-linear association between environmental, social, governance, and development (ESGD) factors and subjective wellbeing, showing that ESGD impacts on happiness follow threshold dynamics rather than a simple linear pattern—an insight that parallels our investigation of potential non-linearity in the happiness-health relationship.

Beyond behaviors, happiness may exert direct physiological effects that influence NCD etiology. Positive emotional states are associated with lower chronic stress activation—e.g. reduced cortisol output and sympathetic nervous system arousal—and with more optimal autonomic function (e.g., higher heart rate variability), all of which can promote cardiovascular health. A very convincing finding in recent literature is the relationship between psychological health and inflammation. Many studies have demonstrated that when individuals report higher levels of positive affect or life satisfaction, their levels of systemic inflammatory biomarkers, such as C-reactive protein (CRP) and interleukin-6 (IL-6) are lower, and this persisted even when accounting for any depressive symptoms and other possible confounds (23). For instance, a very recent systematic review and meta-analysis of ~95,000 individuals found significant associations between greater levels of positive wellbeing and lower levels of circulating IL-6 and CRP (*r* ≈ −0.05 to −0.06), thus demonstrating a small, yet considerable anti-inflammatory effect (24). Chronic systemic inflammation, known to promote the emergence of atherosclerosis, diabetes, and arthritis, is implicated in a plethora of NCDs, and less or absent inflammation diminishes those risks. Furthermore, optimistic or happy individuals demonstrate better metabolic profiles (e.g., lipid and glucose) and immune responses that provide some protection against disease. Importantly, it appears that the biological effects of wellbeing are not simply due to the absence of depression; positive mental health has specific biological effects.

For one longitudinal research study of older adults, both forms of wellbeing—hedonic wellbeing (pleasure in life) and eudaimonic wellbeing (meaning in life)—predicted lower levels of inflammatory biomarkers at follow-up, including latent variables for both variables (the items measured) over time, and independent of depressive symptoms, socio-economic status, and baseline health conditions (23). This reinforces the idea that cultivating happiness might directly “get under the skin” to influence physiology in health-promoting ways.

Several empirical studies have explored and supported the association between happiness and non-communicable diseases. Recent evidence suggests that higher life satisfaction predicts superior long-term health.

In a Canadian population cohort of more than 73,000 adults tracked for 6 years, Rosella et al. (25) observed a gradient: participants least satisfied with life faced markedly greater risks of incident chronic disease and death than their very satisfied peers. After adjusting for demographics, baseline health, and behaviors, the most dissatisfied group exhibited a 70% higher hazard of developing a new non-communicable disease and a roughly 60% higher hazard of mortality. The findings position low life satisfaction as an independent predictor of NCD burden and survival.

Rozanski et al. (20) synthesized 15 longitudinal cohorts, involving ~230,000 participants and demonstrated that optimism is a strong predictor of cardiovascular health. The optimists had a 35% reduced risk of major cardiac events (myocardial infarction, stroke, or cardiac death) and significantly lower all-cause mortality risk than the pessimists, demonstrating the prognostic capability of psychological outlooks across populations and follow-ups. Perhaps most interestingly, the relationship was independent of traditional risk factors and depression, suggesting that there is some independent contribution of optimism to heart health. They found a dose-response relationship: more optimism was associated with less cardiovascular risk. The authors also considered different populations (six different countries), bolstering the idea that the happiness–heart disease relationship extends beyond Western cultures. This meta-analysis also pointed out potential mechanisms, indicating that optimistic individuals tended to exhibit healthier metabolic profiles, lower markers of inflammation, and to engage more in preventive health behaviors; these factors undoubtedly account for some of the reported decreased risks.

Cardiovascular disease tops global NCD mortality. Analyses of nearly 500 000 UK Biobank participants showed that greater happiness and life satisfaction predicted less CVD—particularly stroke, heart failure, and myocardial infarction. Those reporting highest wellbeing faced 40–60% lower risk, partly mediated by healthier behaviors and reduced systemic inflammation, according to study findings (21). Despite some of this relationship, we were surprised to find that a direct protective effect of wellbeing persisted, which suggests some other mechanism, either biological or psychosocial. Overall, the results highlight the potential for enhancing psychological wellbeing as a complimentary strategy for prevention of cardiovascular diseases (21).

Positive wellbeing outcomes extend beyond just cardiovascular health, and are linked to lower type 2 diabetes development, and better glycemic control for those with diabetes. In older adults, higher life satisfaction is associated with lower incidence of stroke, arthritis and cognitive decline. In a 2024 UK developing countries longitudinal study, the authors found that one unit decrease in poor psychological wellbeing increased the risk for several non-communicable diseases (NCDs)—including heart disease, stroke, arthritis, and Alzheimer's each—and increased comorbidity number–regardless of obesity (26). Collectively, the authors' findings suggest that psychological wellbeing could be a unique, modifiable target for chronic disease prevention.

To conclude, current literature provides strong support for the happiness–NCD nexus. Biological studies suggest plausible pathways through which positive psychological wellbeing may affect behaviors, endocrine and immune function, and eventually chronic disease risk.

Taken together, the literature converges on a consistent association between higher subjective wellbeing and healthier behaviors, lower systemic inflammation, and reduced risks of chronic disease and mortality, including evidence from longitudinal cohorts and meta-analyses. At the same time, findings are not uniformly consistent, and several reviews urge caution about treating positive wellbeing as a definitive “health asset.” Moreover, theoretical and empirical arguments suggest non-linearity (potential thresholds and even “too much of a good thing”) implying that marginal health returns to happiness may depend on baseline levels and context; these tensions motivate our threshold-based approach.

### 2.2 The relationship between non-communicable diseases and behavioral, demographic, environmental, and economic/institutional factors

The lifestyle and behavioral drivers behind the rapid rise in NCD prevalence are challenging us all. They stem from complex interactions among individual behaviors, demographic shifts, environmental pressures, and socio-economic conditions—dynamics that are becoming increasingly difficult to manage in the context of rapid modernization. These exploratory determinants include behaviors such as alcohol consumption because drinking particular kinds of alcohol affect the prevalence of disease (27–29). Alcohol consumption is implicated directly in a range of conditions that contribute to NCDs such as cancers, cardiovascular diseases, liver cirrhosis, and pancreatitis, causing ~3–6% of deaths attributed to NCDs globally (27–29). Epidemiological evidence suggests a dose–response association that explains that for every additional increase of per capita consumption of alcohol among a population could equate to an increase in the number of respective thromboembolic hypertensive disorders, strokes, some cancers, and sudden cardiac ischemia, incidences (27, 28). Studies by Griswold et al. (28) indicated that at least as of 2016, alcohol does rank among the top 10 risk factors globally as a primary source of death and disability as seventh among 329 risk factors. The number of alcohol users with consumption levels above low and fatally high, means that there is an urgent requirement to do more if we are to reduce alcohol consumption created by far too many choices for the prompting of excessive use and fatal consequences as promoted throughout our societies and communities and even legislated by governments (28).

Obesity is another major behavioral determinant and is also closely allied with risk for a range of chronic NCDs such as type 2 diabetes, cardiovascular diseases, and some cancers (30–32). Obesity prevalence rose dramatically throughout the world and has doubled in many populations and even quadrupled in others, due to decreases in physical activity and changes in dietary intake (31, 32). Meta-analyses show the significant effects of obesity, including an 80% increased risk of type 2 diabetes and a higher risk of hypertension and dyslipidemia with obesity in overweight individuals (30). It is estimated that four million deaths in 2015 alone were directly associated with obesity, making it one of the foremost risk factors for NCDs (31). Since obesity can shorten life expectancy significantly, lifestyle and dietary changes are vital in solving the global NCD burden (32).

Demographic changes, especially urbanization, have enormous effects on NCD trends. Urbanization affects population health to large extents in conflict to effects of urban living on lifestyle risk factors relating to NCDs, including sedentary behaviors, obesity, and diets (33–35). Urban populations are observed as having a greater burden of diabetes, hypertension, and hyperlipidemia comparative to rural populations based on food accessibility, consumption of processed foods and lifestyle behaviors (35). A global analysis of 173 countries found that greater urbanization is positively associated with both BMI and cholesterol levels. The study further shows that low- and middle-income countries experiencing rapid urban growth display diverse trends in weight-related non-communicable diseases over time (34). Urbanization can have indirect effects on the burden of non-communicable diseases (NCDs) and should therefore be considered in the context of public health interventions, and urban design to mitigate or reduce the burden on NCDs (33, 35).

Air quality, in particular air pollution (PM2.5), is an important environmental factor that has lasting effects on morbidity and mortality of NCDs. PM2.5 exposures are recognized as aggravators for both cardiovascular and respiratory ailments as it has all-year-around exposures and has been estimated as one of the leading risks for mortality worldwide at an estimated 4.2 million NCD-related deaths each year (36, 37). As exposures are higher (for every 10 μg/m^3^) for PM2.5, they have been associated with increased risk for cardiovascular events and lung cancers (37). Placing new models into consideration, PM2.5 air pollution may contribute to over 8 to 9 million premature deaths, revealing the extremes to which air pollution has consequences on human lives (36, 37).

Economic and institutional factors play an important role in health outcomes for NCDs since they affect the health care system and social determinants of health. The quality of governance and specifically the control of corruption are important for a nation's ability to reduce and manage NCDs. A nation with high levels of corruption will have reduced effectiveness of public health practices, will waste resources and money on low quality health care, and this will increase the burden of NCDs on that nation (38, 39). Cross-national research has shown strong associations between lower levels of corruption and lower levels of premature NCD mortality, which included lower age-adjusted mortality rates from cardiovascular diseases, cancers, diabetes, and respiratory diseases (39). Distinctly, weak governance and corruption is associated with a higher prevalence of NCDs and highlights the importance of institutional quality for public health (38).

Health spending is also a significant variable for NCD outcomes. Increased healthcare spending, although a challenging health equity variable, generally results in better prevention, early diagnosis, and effective management of patients with chronic conditions, therefore reducing the burden of disease (40–42). For example, a study of the European Union from 2000 to 2019 found that increases in spending on healthcare were associated with measurable reductions in NCD-related disability-adjusted life years (DALY) related to CVD and cancer- testified by the study of Torres et al. (42). While the latter study emphasizes the importance of health spending, it is important to note that increased spending effectiveness is dependent on strength of governance; wasted health spending or corruption results in diminishing health returns on health spending (40, 41).

Finally, economic development as measured by GDP per capita is an incredibly powerful influence on non-communicable disease patterns. Economic development can also catalyze the beginning of the epidemiological transition, decreasing infectious disease rates and increasing rates of non-communicable diseases (NCD). Middle-income and high-income countries have increasingly and greatly increased incidences of obesity, diabetes, and cardiovascular disease possible from their new lifestyles with wealthier populations (43). Even high-income countries can see an increasing incidence of NCD in their populations while having a lower disease and mortality rate from NCD with a strong public health system (44, 45). Multiple lower-middle-income countries are now concurrently experiencing rapid increases in NCD burden yet do not have adequate health care infrastructure, highlighting the importance of being proactive early in time to develop a strong health strength as they develop economically (44). Overall, these lessons emphasized the need to invest in stronger health systems and preventative strategies while economies grow, as a part of effectively addressing expected pressures around NCDs (43–45).

As a concluding synthesis of the literature presented in this subsection, the evidence converges on several regularities. Harmful alcohol use, excess adiposity, and fine-particulate exposure (PM_2.5_) are consistently linked to higher NCD morbidity and premature mortality and are amenable to policy intervention. Higher (and better targeted) health expenditure and stronger governance generally associate with lower NCD mortality, though efficiency varies across settings. By contrast, the effects of urbanization and economic development are context-dependent: early-stage urbanization often elevates risk, whereas mature, well-serviced urban environments can be neutral or protective; similarly, mortality returns to GDP per capita are more evident in higher-capacity or higher-happiness contexts. Contradictory findings largely reflect measurement heterogeneity, model specification and endogeneity concerns, and plausible non-linearities or thresholds. These convergences and tensions motivate our regime-dependent, threshold-based framework and the hypotheses stated in Section 2.3.

### 2.3 Literature gap and research contribution

Although a sizable body of work links subjective wellbeing with favorable health markers, most empirical studies impose linearity (treating happiness as a uniformly acting predictor) and seldom probe for thresholds or regime shifts in large cross-country panels. This leaves unclear whether marginal gains in happiness are equally health-productive at all baseline levels, or whether benefits emerge only after a minimum wellbeing “tipping point.” Such linear specifications risk policy misdirection if non-linearities are present. Moreover, prior evidence is often confined to single countries or short horizons and rarely examines dynamic bidirectionality between happiness and mortality at scale.

We address these gaps by (i) assembling a balanced global panel of 123 countries (2006–2021) and (ii) estimating a Panel Smooth Transition Regression (PSTR) that uses the Life-Ladder index as the transition variable, allowing coefficients—on happiness and on key behavioral, environmental, demographic, and institutional covariates—to vary across regimes separated by an empirically estimated threshold. This design identifies a single, policy-salient cutoff at ≈2.7 Life-Ladder points; above that level, increases in happiness are associated with statistically significant reductions in premature NCD mortality, while below it the effect is negligible. We further complement the non-linear estimates with panel-VAR tests and impulse-response analyses to characterize dynamic feedbacks between happiness and mortality. Together, these features move beyond linear models, quantify the threshold at which happiness becomes a population health asset, and clarify regime-dependent covariate effects.

Compared with earlier studies, our contribution is threefold: (1) first global-scale, threshold-focused test of the happiness–NCD nexus using PSTR, rejecting parameter constancy and documenting a two-regime structure; (2) explicit estimation of the threshold (≈2.7) and of regime-specific elasticities, showing no evidence (within the observed range) of a “too-much-happiness” penalty; and (3) demonstration of bidirectional dynamics (happiness ↔ NCD mortality) that help explain virtuous cycles in population health. These advances sharpen theory and provide operational guidance for governments debating where and how to target wellbeing to yield measurable survival gains.

Hypotheses:

H1: The relationship between national happiness and premature NCD mortality is non-linear, with a threshold in Life-Ladder scores beyond which higher happiness reduces mortality.
H2: Above the estimated threshold, increases in happiness are associated with statistically significant declines in NCD mortality; below the threshold, the effect is null.
H3: Happiness and NCD mortality exhibit bidirectional (Granger) causality with an adverse sign (higher happiness forecasts lower mortality and vice versa).

## 3 Methodology

### 3.1 Data

Happiness, representing psychological wellbeing, is measured using the Gallup World Poll's *Life Ladder* question: “*Please imagine a ladder, with steps numbered from 0 at the bottom to 10 at the top. The top represents the best possible life for you, and the bottom the worst possible life. On which step of the ladder would you say you personally feel you stand at this time?”* Individual responses (ranging from 0 to 10) are averaged at the country level to produce a national measure of subjective wellbeing (46). Averaged country-year scores provide a standardized, cross-national indicator captured in the *World Happiness Report*. This subjective metric aligns with both the WHO's holistic concept of health (47, 48) and the UN's development goals (49, 50), and reflects more than economic performance. Evidence links higher *Life Ladder* scores with healthier behaviors and reduced physiological stress (11, 51, 52). They also respond to broader societal events such as economic crises erode happiness (53), while climate finance in Africa is shown to enhance life satisfaction, even accounting for indirect pathways such as political stability and natural resource rents (52).

Following Hansen (54), we construct a balanced panel of 123 countries observed over the period 2006–2021. The data are sourced from the World Health Organization, World Development Indicators, and the Gallup World Poll (see Table 1). The sample period is determined by the availability of NCD mortality data, which extends up to 2021 based on WHO data.

**Table 1:** Descriptive statistics.

**Series**	***N***	**Obs**.	**Mean**	**Std**.	**Min**.	**Max**.	**Source**
NCD mortality rate	123	1,968	19.03	7.007	7.50	43.10	World Health Organization
Life ladder	123	1,968	5.45	1.185	2.18	7.97	Gallup World Poll
Alcohol consumption	123	1,968	5.84	4.272	0.00	18.39	World Health Organization
Body mass index	123	1,968	17.36	9.1979	1.00	41.48	World Health Organization
Urban population	123	1,968	60.03	22.43	15.14	100	World Development Indicator
Air pollution	123	1,968	27.15	1.7415	4.75	107.14	World Development Indicator
Control of Corruption	123	1,968	−0.02	1.0499	−1.67	2.44	World Development Indicator
Health expenditure	123	1,968	1292.55	2040.87	8.42	11999.09	World Development Indicator
GDP per capita	123	1,968	15582.2	21537.1	189.3	133711.8	World Development Indicator

To ensure data harmonization across countries, we applied the Amelia II multiple imputation method to handle missing values while preserving cross-country and temporal variation. Relevant variables were log-transformed to reduce skewness and ensure comparability of scales. Finally, lagged values of the explanatory variables were used in modeling to capture dynamic effects and address potential simultaneity.

Table 1 reports descriptive statistics, including the minimum, maximum, mean, and standard deviation for the selected raw variables. The mortality rate, defined as the percentage of deaths due to non-communicable diseases among individuals aged 30–70, shows considerable cross-country variation. On average, 19% of deaths are attributable to these causes, with country-year values ranging from 7.5% in Switzerland (2021) to 43% in Uzbekistan (2006) over the study period.

Descriptive statistics show that the average Life Ladder score across the 123 countries during 2006–2021 is 5.45, with a minimum of 2.18 and a maximum of 7.97. Average per capita alcohol consumption, measured over a calendar year, is 5 liters, with the highest observed value being 17 liters. The prevalence of obesity, defined as the share of adults with a body mass index (BMI) of 30 or more, averages 17.4%, with a relatively high standard deviation of around 10 percentage points, indicating considerable variability among countries.

The urban population variable, representing the proportion of individuals living in urban areas relative to the total population, has a global average of 60%. However, there is substantial variation across countries, with values ranging from 15% in Malawi (2006) to complete urbanization at 100% in Singapore (2006). Air pollution exposure is quantified as the average annual level of fine particulate matter (PM2.5)—airborne particles with a diameter of < 2.5 microns that can penetrate deep into the lungs and contribute to serious health issues. The average concentration is 27.15 μg/m^3^, with the highest recorded level reaching 107.14 μg/m^3^ in Niger (2015) across the sample.

The variable control of corruption reflects the degree to which public authority is used for private benefit, encompassing both minor and large-scale corruption, as well as undue influence by elites or private actors over state institutions. It is a standardized index, typically ranging from ~-2.5 to 2.5, where higher scores indicate stronger institutional integrity. The global average of −0.02 suggests that many countries face ongoing governance challenges. In several instances, scores fall below −1.5, such as in Myanmar (2010), indicating serious governance challenges, whereas a few countries, like Denmark (2009), exhibit strong governance with scores approaching 2.5.

Regarding health-related expenditure, current health spending per capita averages approximately USD 1,300, with values ranging from as low as USD 9 in Ethiopia (2006) to as high as USD 12,000 in the United States (2021). Lastly, GDP per capita averages USD 16,000 across the selected countries, though substantial disparities are evident between the lowest and highest-income nations.

### 3.2 Empirical modeling

From an empirical standpoint, unlike other metrics of subjective wellbeing, the Life-Ladder score is the only indicator consistently available as a balanced panel across 123 countries over the study period. Moreover, when evaluated as a transition variable during preliminary model selection, it yielded the highest LM-F statistic (8.852) and indicated the presence of a single smooth transition function. These findings suggest that variation in national happiness most effectively captures the non-linear regimes through which other covariates, such as alcohol use, obesity, pollution, and governance exert their influence on premature NCD mortality (9).

We adopt the Panel Smooth Transition Regression (PSTR) framework proposed by González et al. (55), which provides a robust and flexible approach for modeling non-linearities and cross-sectional heterogeneities in panel data. The PSTR specification allows regression coefficients to vary smoothly and boundedly with respect to an observable transition variable, enabling the detection of gradual shifts between distinct regimes without imposing abrupt structural breaks. While alternative approaches such as quantile regression offer valuable insights into distributional heterogeneity (56, 57) and fixed-effects quantile models further account for unobserved heterogeneity (56, 58), these methods focus on estimating conditional effects at specific quantiles rather than modeling endogenous regime transitions. Consequently, PSTR is better suited to capturing the smooth, regime-dependent non-linear dynamics central to our empirical analysis.

The basic PSTR model with two extreme regimes is defined as:


(1) $$Y_{it}=\mu_{i}+\lambda_{t}+\beta_{0}^{'}X_{it}+\beta_{1}^{'}X_{it}g\left(Q_{it};\gamma ,c\right)+u_{it}$$

For *i* = *1 ,…,N* and *t* = *1 ,…,T*, where N and T represent the cross-sectional and temporal dimensions of the panel, respectively, the model is specified with *Y*_*it*_ as the dependent variable. Here, *Y*_*it*_ is a scalar outcome of interest, while *X*_*it*_ denotes a k-dimensional vector of time-varying explanatory variables. The terms μ_*i*_ and λ_*t*_ capture unobserved individual-specific and time-specific fixed effects, respectively, accounting for heterogeneity across units and common shocks over time. The error term *u*_*it*_ is assumed to be well-behaved, representing the idiosyncratic disturbances not explained by the model.

The transition function *g*(*Q*_*it*_; γ, *c*) in Equation 1 is a continuous function of the observable transition variable *Q*_*it*_ and is normalized to lie within the interval [0,1].

#### 3.2.1 Step 1: determining the functional form of transition function

We adopt the logistic function specification for the transition function *g*(*Q*_*it*_; γ, *c*) because it allows for a smooth and continuous transition between regimes, which is particularly suited for capturing gradual structural changes rather than abrupt shifts. This choice aligns with the theoretical expectation that the effect of explanatory variables on the dependent variable evolves progressively as the transition variable crosses certain thresholds, rather than changing instantaneously.


(2) $$\begin{array}{r} g\left(Q_{it};\gamma ,c\right)=={\left(1+\exp\left(-\gamma \overset{m}{\underset{j=1}{\prod }}\left(Q_{it}-c_{j}\right)\right)\right)}^{-1}\\ with\;\gamma >0\;and\;c_{1}<c_{2}<\ldots <c_{m} \end{array}$$

where $c={\left(c_{1},c_{2},\ldots ,c_{m}\right)}^{\prime }$ is an m-dimensional vector of location parameters and the slope parameter γ determines the smoothness of the transitions.

#### 3.2.2 Step 2: model specification

In the panel data context, the specification stage entails testing for parameter homogeneity, selecting the transition variable *Q*_*it*_ and, if homogeneity is rejected, identifying the appropriate functional form of the transition mechanism, specifically determining the optimal value of *m* in Equation 2.

The PSTR model (1) with (2) can be reduced to a homogeneous model by imposing either H0: γ= 0 or H0: $\beta_{1}^{'}=0$. The associated tests are non-standard, as the PSTR model under the null hypothesis includes unidentified nuisance parameters. Next, we test for parameter homogeneity using the null hypothesis H: γ = 0. To overcome the identification problem, the transition function in Equation 1 is replaced by its first-order Taylor series expansion around γ = 0. This reparameterization leads to the formulation of following auxiliary regression used to detect non-linearity.


(3) $$\begin{array}{l} Y_{it}=\mu_{i}+\beta_{0}^{\prime *}X_{it}+\beta_{1}^{\prime *}X_{it}Q_{it}+\ldots +\;\beta_{m}^{\prime *}X_{it}Q_{it}^{m}+u_{it}^{*} \end{array}$$

where the parameters $\beta_{1}^{*},\beta_{2}^{*},\ldots ,\;\beta_{m}^{*}$ are multiples of γ and $u_{it}^{*}=u_{it}+r_{m}\beta_{1}^{\prime }X_{it},$ where *r*_*m*_ is the reminder of the Taylor expansion. Consequently, testing the null hypothesis *H*_0_:γ= 0 in Equation 1 is equivalent to testing $H_{0}:\beta_{1}^{*}=\beta_{2}^{*}=\ldots =\beta_{m}^{*}=0$. Under the null hypothesis, the LM-F statistic is computed as *LM*_χ_(*TN*−*N*−*k*−*mk*)/(*TNmk*), which approximately follows an *F*(*mk, TN -N – k-mk*) distribution. In our case, the LM-F test statistic corresponding to Life Ladder is substantially higher than for the other variables, indicating that it is the most suitable choice for the transition function in the panel smooth transition regression model.

Next, the homogeneity test can also serve as a basis for selecting the appropriate order *m* of the logistic transition function specified in Equation 2. The sequential testing procedure involves first estimating the auxiliary regression in Equation 3 with m = 3, and testing the null hypothesis $H_{0}^{*}=\beta_{3}^{*}=\beta_{2}^{*}=\beta_{1}^{*}=0$. If it is rejected test $H_{03}^{*}=\beta_{3}^{*}=0\;;H_{02}^{*}=\beta_{2}^{*}=0|\beta_{3}^{*}=0\;$and $H_{01}^{*}:\;\beta_{1}^{*}=0|\beta_{3}^{*}=\beta_{2}^{*}=0\;$. Based on the results of the sequential testing procedure, the value *m*=*2* is selected if the null hypothesis $H_{02}^{*}\;$exhibits the strongest evidence against it. Otherwise, if the rejection is not supported for $H_{02}^{*}\;,\;$the model proceeds with *m*=*1* as the appropriate order for the logistic transition function. In our case, the LM-F statistic consistently support the specification with one transition function across all candidate variables.

#### 3.2.3 Step 3: model estimation

Next, the estimation of the parameter $\theta ={\left(\beta_{0}^{\prime },\;\beta_{1}^{\prime }\;,\;\gamma ,\;c\right)}^{\prime }\;$ in the PSTR model specified in Equation 1 is carried out using a combination of the fixed effects estimator and non-linear least squares (NLS). To begin, the individual-specific effects μ_*i*_ are removed by applying the within transformation, which involves subtracting individual means from the data. NLS is then performed on the transformed dataset to obtain consistent parameter estimates.

#### 3.2.4 Step 4: model evaluation

In the final evaluation stage, the adequacy of the estimated model is assessed through a series of misspecification tests. These include testing for parameter stability, no remaining heterogeneity and no autocorrelation in the errors. Furthermore, we conduct a sensitivity analysis by incorporating an alternative external variable as a transition variable candidate, health-adjusted life expectancy (HALE) as a proxy measure for happiness. The estimated coefficients obtained from this alternative specification are then compared with the baseline results to evaluate the robustness of our findings.

To further investigate the joint dynamic interactions among the variables, the study adopts a Panel Vector Autoregression (PVAR) framework. PVAR models are particularly well-suited for examining the transmission of shocks across units over time. By integrating features from both traditional time-series VAR models and dynamic panel data structures, PVAR enables the analysis of interdependencies among multiple variables within a panel setting. This study employs the Panel Vector Autoregression (PVAR) approach and conducts estimation within a Generalized Method of Moments (GMM) framework to address potential endogeneity and dynamic bias (59). The model specification follows the implementation described by Abrigo and Love (60), wherein variables are first-differenced to remove unit-specific fixed effects. In this specification, only lagged values of the dependent variables are included as regressors, and the estimation is conducted using the R programming environment. The estimated model is given as follows:


(4) $$\begin{array}{l} Y_{it}=A_{1}Y_{i,t-1}+A_{2}Y_{i,t-2}+\ldots +A_{p}Y_{i,t-p}+\epsilon_{it} \end{array}$$

where *Y*_*it*_ is a *K* × 1 vector of first differenced transformed dependent variables; *A*_1_, *A*_2_, …., *A*_*p*_ are *K* × *K* matrices of parameters to be estimated and ϵ_*it*_ is a *K* × 1 vector of serially uncorrelated idiosyncratic errors, respectively. The autoregressive structure of the PVAR model allows each endogenous variable to enter the system with its own lag, capturing dynamic interdependencies over time. A key advantage of the PVAR framework is that it treats all variables as endogenous, thereby explaining the simultaneous modeling of feedback effects between NCD mortality rates and multiple dimensions of subjective wellbeing. Beyond this, the methodology offers several econometric strengths. Notably, it addresses potential endogeneity concerns that frequently arise in panel data settings. Moreover, the use of Generalized Method of Moments (GMM) estimators, enables efficient estimation in panels characterized by a large cross-sectional dimension (N) and a relatively short time dimension (T).

The panel VAR approach involves several estimation steps, beginning with the selection of the optimal lag length, an important consideration that influences both the estimated coefficients and the resulting impulse response functions. Selecting too many lags may reduce the model's degrees of freedom and lead to overfitting, while too few lags may fail to capture the full dynamic structure, resulting in omitted variable bias. To address this, the optimal lag order is determined using the Consistent Model and Moment Selection Criteria (MMSC) proposed by Andrews and Lu (61), which are tailored for GMM-based panel VAR estimation. These criteria are adaptations of well-known information criteria—Akaike Information Criterion (AIC), Bayesian Information Criterion (BIC), and Hannan–Quinn Information Criterion (HQIC)—to the context of panel data and GMM estimation. Unlike their likelihood-based counterparts, the MMSC are derived from Hansen's J-statistic and apply penalty terms that account for both sample size and the number of overidentifying restrictions. The optimal lag length is selected as the value that minimizes the respective AIC, BIC, or HQIC.

Once the optimal lag length is selected, it is essential to verify the stability of the panel VAR model, as empirical results cannot be meaningfully interpreted if the system is unstable. Stability ensures that the Impulse Response Functions (IRFs) converge over time following a shock. According to Lutkepohl (62) and Hamilton (63), a VAR model is considered stable if all the eigenvalues (moduli) of the companion matrix lie strictly within the unit circle. This condition guarantees that the panel VAR is invertible and admits a vector moving-average (VMA) representation of infinite order, thereby enabling a well-defined and interpretable analysis of dynamic responses through IRFs.

After establishing the optimal lag length and confirming the model's stability, the next step involves conducting Granger causality tests to assess the predictive relationships among the variables. Specifically, the test evaluates whether the past values of one variable contain information that helps forecast another variable beyond what is contained in its own past. Formally, variable X is said to Granger-cause variable Y if, for all s>0, the mean squared error (MSE) of forecasting *Y*_*t*+*s*_ based on the history of both (*Y*_*t*−1_, *Y*_*t*−2_, ……, *X*_*t*−1_, *X*_*t*−2_, ….) is lower than the MSE of a forecast using only the past values of (*Y*_*t*−1_, *Y*_*t*−2_, ….). Similar to standard time-series VARs, Granger causality in the panel VAR context can be assessed using Wald tests, which evaluate the joint statistical significance of the coefficients on the lagged values of one or more variables in the relevant equation (63).

Finally, to explore the dynamic response of one variable to innovations in another while controlling for contemporaneous effects, impulse response function (IRF) analysis is conducted. A key advantage of the VAR framework lies in its ability to trace the impact of orthogonal shocks—capturing how a shock to one variable affects another, holding all other shocks constant. To achieve this, shocks are orthogonalized using the Cholesky decomposition of the estimated error covariance matrix. In this way, PVAR enables a clear and interpretable representation of the temporal transmission mechanisms within the system.

## 4 Results

### 4.1 Subjective wellbeing and non-communicable disease mortality rate: panel smooth transition regression (PSTR)

Prospective studies consistently find that positive psychological wellbeing (PWB) leads to better physical health outcomes (64). Subjective wellbeing, which encompasses life satisfaction, positive affect, and optimism has been linked to a reduced risk of cardiovascular disease (65). A comprehensive meta-analysis of 35 prospective studies reported a significant association between PWB and decreased mortality risk (66). While several studies support the link between psychological wellbeing and health, some findings remain inconsistent, prompting healthy skepticism about PWB's standing as a true “health asset” (67). As PWB starts gaining recognition alongside other well-known determinants like poverty, education, discrimination, and social cohesion (68), resolving these ambiguities becomes essential. Unless we clearly understand why some evidence diverges, it will be hard to justify incorporating positive wellbeing into public health policies and practice (69).

To investigate whether psychological wellbeing (PWB) influences non-communicable disease (NCD) mortality and to explore the nature of this relationship, including potential heterogeneity across countries, we employ a panel smooth transition regression (PSTR) model. For each country *i* in year *t*, we use the Life Ladder score (*L*_*i, t*_) as a proxy for PWB. In addition, we incorporate alcohol consumption (*D*_*i, t*_) and obesity prevalence (BMI ≥ 30) (*BMI*_*i, t*_) as behavioral indicators, the urbanization rate (*N*_*i, t*_) as a demographic factor, air pollution (PM2.5exposure) (*P*_*i, t*_) as an environmental measure, and control of corruption (*CC*_*i, t*_), per capita health expenditure (*H*_*i, t*_), and GDP per capita (*Y*_*i, t*_) as proxies for institutional and economic conditions. To account for the dynamic nature of the panel data, we include the lagged value of the NCD mortality rate (*NCD*_*i, t*−1_) as a regressor.

All variables, except control of corruption, are log-transformed to ensure consistency in scale across indicators. The model also controls for both country-fixed effects and time-fixed effects to account for unobserved heterogeneity and global macroeconomic trends affecting mortality.

Following Gonzalez et al. (70) and Hu and Schiantarelli (71), we begin by conducting the LM-F test for parameter homogeneity, using each regressor (lagged Life Ladder, alcohol consumption, obesity prevalence, urbanization rate, air pollution, control of corruption, health expenditure, and GDP per capita) as a potential transition variable. As reported in Table 2, the null hypothesis of homogeneity is strongly rejected for all eight potential transition variables, with *p*-values equals zero. Notably, the LM-F test statistic corresponding to Life Ladder is substantially higher than for the other variables, indicating that it is the most suitable choice for the transition function in the panel smooth transition regression model.

**Table 2:** Homogeneity tests.

**Transition variable**	**Life ladder (L_i, (*t*−1)_)**	**Alcohol consumption (D_i, (*t*−1)_)**	**Body mass index (BMI_i, (*t*−1)_)**	**Urban population (N_i, (*t*−1)_)**
*Ho: r = 0* vs. *H_1_: r = 1*	8.852 (0.00)	2.518 (0.00)	3.284 (0.00)	6.266 (0.00)
Transition variable	Air pollution (*P*_*i*, (*t*−1)_)	Control of corruption (*CC*_*i*, (*t*−1)_)	Health expenditure (*H*_*i*, (*t*−1)_)	GDP per capita (*Y*_*i*, (*t*−1)_)
*Ho: r = 0* vs. *H_1_: r = 1*	3.283 (0.00)	4.523 (0.00)	4.071 (0.00)	5.199 (0.00)

The table reports the LMF test statistic for homogeneity, along with the corresponding *p*-values (in parenthesis), from the panel regression of non-communicable diseases mortality rate on lagged life ladder, alcohol consumption, urban population, air pollution, control of corruption, health expenditure, and GDP per capita. The analysis is based on a balanced panel of 123 countries covering the period 2006–2021.

Next, we adopt a sequential testing approach to assess the number of transition functions required. After rejecting the null hypothesis of linearity, we test whether the data are better explained by a single transition function (H0: *r* = 1) or by at least two transition functions (H0: *r* = 2). As shown in Table 3, the LM-F statistic consistently support the specification with one transition function across all candidate variables. This result further supports the selection of Life Ladder as the transition variable in the model.

**Table 3:** Sequence of homogeneity tests for selecting order r of transition function.

**Transition variable**	**Life ladder (L_i, (*t*−1)_)**	**Alcohol consumption (D_i, (*t*−1)_)**	**GDP per capita (Y_i, (*t*−1)_)**	**Urban population (N_i, (*t*−1)_)**
*Ho: r = 0* vs. *H_1_: r = 1*	8.852 (0.00)	2.518 (0.00)	5.199 (0.00)	6.266 (0.00)
*Ho: r = 1* vs. *H_1_: r = 2*	1.083 (0.357)	1.302 (0.553)	0.899 (0.556)	1.670 (0.226)
*Ho: r = 2* vs. *H_1_: r = 3*	–	–	–	–
**Transition variable**	**Air pollution (***P*_*i*, (*t*−1)_)	**Control of corruption (***CC*_*i*, (*t*−1)_)	**Health expenditure (***H*_*i*, (*t*−1)_)	**Body mass index (***BM*_*i*, (*t*−1)_)
*Ho: r = 0* vs. *H_1_: r = 1*	3.283 (0.00)	4.523 (0.00)	4.071 (0.00)	3.284 (0.00)
*Ho: r = 1* vs. *H_1_: r = 2*	1.059 (0.386)	1.080 (0.361)	1.224 (0.151)	0.928 (0.607)
*Ho: r = 2* vs. *H_1_: r = 3*	–	–	–	–

For each transition variable, the testing procedure proceeds sequentially. First, a linear specification (*r* = *0)* is tested against a single-transition model (*r* = *1*). If the null hypothesis is rejected, the single-transition model is then tested against a double-transition model (*r* = *2*), and so on. The process continues until the null hypothesis of no additional transition is no longer rejected. Under the null hypothesis, the LMF statistic follows an asymptotic *F[1,TN–N–(r*+*1)]* distribution. Corresponding *p*-values are reported in parentheses.

Thus, we proceed to estimate the following Panel Smooth Transition Regression (PSTR) model:


(5) $$\begin{array}{r} NCD_{i,t}=\mu_{i}+\lambda_{t}+\alpha_{01}\;NCD_{i,\left(t-1\right)}+\beta_{01}L_{i,\left(t-1\right)}+\beta_{02}D_{i,\left(t-1\right)}\\ +\beta_{03}BMI_{i,\left(t-1\right)}+\beta_{04}N_{i,\left(t-1\right)}+\beta_{05}P_{i,(t-1)}+\beta_{06}CC_{i,(t-1)}\\ +\beta_{07}H_{i,(t-1)}+\beta_{08}Y_{i,(t-1)}+(\alpha_{11}\;NCD_{i,\left(t-1\right)}+\beta_{11}L_{i,\left(t-1\right)}\\ +\beta_{12}D_{i,\left(t-1\right)}+\beta_{13}BMI_{i,\left(t-1\right)}+\beta_{14}N_{i,\left(t-1\right)}+\beta_{15}P_{i,\left(t-1\right)}\\ +\beta_{16}CC_{i,\left(t-1\right)}+\beta_{17}H_{i,\left(t-1\right)}+\beta_{18}Y_{i,\left(t-1\right)})g\left(L_{i,\left(t-1\right)};\gamma ,\;c\right)+u_{it} \end{array}$$

where λ_*t*_ denotes the fixed-time effects, and


(6) $$\begin{array}{l} g\left(L_{i,\left(t-1\right)};\gamma ,\;c\right)={\left(1+\exp\left(-\gamma \left(L_{i,\left(t-1\right)}-c\right)\right)\right)}^{-1}with\gamma >\;0 \end{array}$$

Before discussing the estimation results, we assess the adequacy of the two-regime PSTR model using misspecification tests for parameter constancy and remaining non-linearity, considering Life Ladder as the transition variable. As reported in Table A1 in Appendix, the results indicate no evidence of remaining heterogeneity, confirming that the model with a single transition function provides a satisfactory specification.

Parameter estimates are presented in Table 4. We report the regression coefficients for both regimes: one associated with low values of the transition function and the other with high values. The estimated slope of the transition function is 1.673, indicating that the shift from the lower regime, characterized by low subjective wellbeing, to the upper regime, associated with higher wellbeing, is gradual rather than abrupt. This smooth transition is also illustrated in Figure A1 in Appendix.

**Table 4:** Estimation results of two-regime PSTR model.

**Variables**	**Low life ladder scale countries**	**High life ladder scale countries**
**Dependent variable: NCD mortality rate** (*NCD*_*i, t*_)		
**Explanatory variables:**		
L.NCD mortality rate (*NCD*_*i, t*−1_)	0.829^***^ (0.042)	0.843^***^ (0.074)
Life ladder (*L*_*i*, (*t*−1)_)	−0.158 (0.144)	−0.429^***^ (0.061)
Alcohol consumption (*D*_*i*, (*t*−1)_)	0.012^**^ (0.006)	0.013^**^ (0.005)
Body mass index (*BMI*_*i*, (*t*−1)_)	0.157^***^ (0.011)	0.063^**^ (0.014)
Urban population (*N*_*i*, (*t*−1)_)	0.141^***^ (0.032)	-−0.499^***^ (0.036)
Air pollution (*P*_*i*, (*t*−1)_)	0.046^***^ (0.022)	0.001 (0.086)
Control of corruption (*CC*_*i*, (*t*−1)_)	−0.032 (0.039)	−0.001 (0.038)
Health expenditure (*H*_*i*, (*t*−1)_)	−0.092^**^ (0.017)	−0.087^**^ (0.014)
GDP per capita (*Y*_*i*, (*t*−1)_)	0.059 (0.156)	−0.120^***^ (0.014)
Location parameter (*c*)	2.719 (3.818)	
Slope parameter (γ)	1.673 (0.582)	

The standard errors for coefficients in parentheses are corrected for heteroskedasticity. ^***^p < 0.01, ^**^p < 0.05.

The discovery that happiness begins to exert a statistically significant, monotonically protective influence on NCD mortality only once national Life-Ladder scores exceed ≈ 2.7 points dovetails with the biopsychosocial pathways highlighted in our literature review. Experimental and longitudinal evidence shows that higher subjective wellbeing improves health behaviors and dampens systemic inflammation - mechanisms that plausibly underpin the 0.43% mortality reduction per 1% rise in happiness observed in the high-wellbeing regime (9, 32). These results are consistent with the longitudinal and meta-analytic findings of Rozanski et al. (20) and Zhong et al. (21), which show that higher life satisfaction and optimism predict lower cardiovascular and all-cause mortality through both behavioral and anti-inflammatory pathways, and do not contradict prior large-scale analyses such as Evans and Soliman (12), which also report a robust positive link between wellbeing and longevity. Likewise, the stronger positive coefficients for obesity and alcohol use when happiness is low echo the behavioral feedbacks documented by Rozanski et al. (20), where insufficient wellbeing predisposes populations to risk-enhancing lifestyles that amplify chronic-disease burden. The persistent positive association between alcohol use and NCD mortality is in agreement with the dose–response patterns reported by Parry et al. (27) and Griswold et al. (28), who found that higher per-capita alcohol consumption increases the risk of multiple chronic conditions, and it does not conflict with recent global burden estimates identifying alcohol among the top behavioral risk factors for premature death. By aligning these empirical regime shifts with established causal pathways, our results extend prior single-country and cohort studies to a global panel context, confirming that population happiness functions not merely as a correlate but as a threshold-dependent driver of premature NCD mortality.

The estimated location parameter is 2.719, which is close to the minimum observed value in the empirical distribution of Life Ladder scores (see Table 1). This suggests that the model identifies countries with low levels of subjective wellbeing as a distinct group, separate from those with moderate or high wellbeing levels.

Interestingly, the transition function statistics reported in Table A2 in Appendix indicates that, prior to 2016, most of the selected countries experienced relatively low levels of subjective wellbeing. This is evident from the small proportion of countries classified in the upper regime during the 2006–2016 period. However, the median value of the transition function remained above 0.6 across the full sample, suggesting a right-skewed distribution. This implies that a smaller group of countries with high levels of subjective wellbeing are primarily responsible for the observed non-linear shifts. In other words, the relationship between non-communicable disease mortality and its explanatory factors tends to change significantly only once a country attains a relatively high level of wellbeing.

Furthermore, the 25th and 75th percentiles of the yearly transition function values indicate that this regime shift is primarily driven by changes in the lower part of the cross-sectional distribution, as evidenced by the 25th percentile rising to around 0.55. This finding supports the presence of time-varying relationships across countries, implying that assuming a fixed classification of countries into low or high subjective wellbeing groups would be inappropriate in this context.

Turning to the estimated regression coefficients, the results show that lagged obesity prevalence has a positive and statistically significant association with non-communicable disease mortality in both low and high subjective wellbeing country groups. However, the effect is more pronounced among countries with lower levels of subjective wellbeing. This finding aligns with the conclusions of the Afshin et al. (31), which reported that high body mass index was responsible for ~4 million deaths worldwide. The elevated mortality risk linked to higher BMI aligns with the meta-analyses of Guh et al. (30) and Afshin et al. (31), which attribute millions of deaths annually to obesity-related cardiometabolic disorders, and contrasts with only a few cohort studies that reported weak or non-significant associations in specific subpopulations.

The rate of urbanization shows a significant and positive association with non-communicable disease mortality in countries with low levels of subjective wellbeing. However, in countries with higher Life Ladder scores, this relationship becomes stronger and shows a negative association. These findings are consistent with existing literature. For instance, Liu et al. (72) introduced the Urbanization–Excess Deaths Elasticity indicator and reported that a one percent increase in urbanization was linked to a 0.5 percent rise in mortality from ischemic heart disease. In contrast, Bandyopadhyay and Green (73), using cross-national panel data, found robust evidence of a negative correlation between crude death rates and urbanization.

Air pollution exhibits a positive and statistically significant impact on non-communicable disease mortality in countries with low Life Ladder scores, while the effect becomes statistically insignificant in countries with higher levels of subjective wellbeing. These findings are consistent with the meta-analysis by Badida et al. (74), which reported significant associations between ambient air pollutants and both short-term and long-term adverse health outcomes in low- and middle-income countries. The attenuation of PM2.5's effect in high-wellbeing countries supports the conclusions of Cohen et al. (36) and Burnett et al. (37) that the most severe health impacts occur in lower-income contexts with weaker environmental regulation, and it diverges from some high-income country evidence where fine-particulate exposure has remained a significant mortality predictor despite advanced pollution controls. In contrast, control of corruption does not show a statistically significant effect on mortality in either group of countries. The absence of a significant relationship between control of corruption and NCD mortality contrasts with the cross-national findings of Holmberg and Rothstein (38) and Botero-Rodríguez et al. (39), which report lower premature mortality in countries with stronger governance, suggesting that in our model such effects may be masked by the inclusion of dominant behavioral and environmental predictors.

Health expenditure emerges as a significant factor in reducing non-communicable disease (NCD) mortality across both groups of countries. This finding is consistent with the meta-analysis by Gallet and Doucouliagos (75), which synthesized results from 65 studies and concluded that healthcare spending has a substantial effect on lowering mortality rates. The consistent protective association of health expenditure with reduced NCD mortality aligns with the findings of Nixon and Ulmann (40), Torres et al. (42), and Iuga et al. (13), who show that greater investment in healthcare improves prevention, early detection, and chronic disease management, and does not contradict prior economic analyses on the mortality benefits of targeted spending.

In contrast, GDP per capita shows a significant inverse relationship with NCD mortality only in countries with higher levels of subjective wellbeing, while its effect is statistically insignificant in countries with lower wellbeing levels. These results are supported by Wang and Wang (76), who found that trends in NCD mortality differ by country type and stage of socioeconomic development, with mortality rates declining as national income rises. The inverse relationship between GDP per capita and NCD mortality in high-wellbeing countries is consistent with the conclusions of Danaei et al. (43) and Gouda et al. (45) that economic growth in developed contexts reduces chronic disease mortality, and it accords with the “economic prosperity and chronic disease paradox” described by Iuga et al. (13), whereby income gains in less-developed settings can coincide with rising NCD risks.

To assess robustness, the original transition variable (Life Ladder Scores) was replaced with Health Adjusted Life Expectancy (HALE) from the WHO Global Health Observatory. HALE is defined as the average number of years a person can expect to live in full health, accounting for mortality and morbidity (86). Preliminary tests indicated that HALE is a suitable transition variable for PSTR estimation (LM-F = 13.69, *p* < 0.001). Sequential HAC-F tests supported a single transition function with two regimes (m = 1: HAC-F = 1.965, *p* = 0.0398; m = 2: HAC-F = 0.917, *p* = 0.5092). Results (Table A4 in Appendix) show that the estimated coefficients across regimes are comparable in sign and magnitude to those obtained with Life Ladder Scores, confirming the robustness of the findings and supporting Life Ladder Scores as the preferred transition variable.

**Figure 1:**
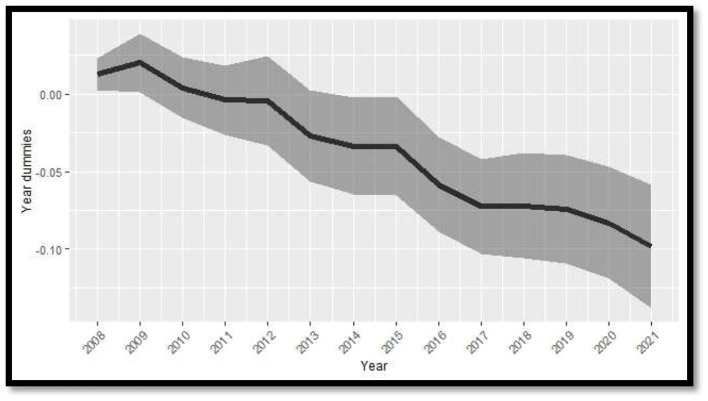
Coefficient estimates of yearly dummies (solid line) with lower and upper bound of 90% confidence intervals based on cluster-robust and heteroskedasticity consistent standard error.

Figure 1 presents the estimated time effects from the PSTR model, along with the lower and upper bounds of the 90% confidence interval, calculated using cluster-robust and heteroskedasticity-consistent standard errors. These time effects are interpreted relative to the baseline year of 2006, which marks the beginning of the effective sample period. The estimates reveal a steady downward trend over time, indicating that, beyond the non-linear impact of the explanatory variables and the transition function based on subjective wellbeing, there has been a general decline in non-communicable disease mortality across countries during the study period.

Our evidence that obesity remains a significant driver of NCD mortality (especially in low-wellbeing countries) accords with the four-million-deaths attribution reported by the Afshin et al. (31), study and with recent longitudinal findings by Putra et al. (26) that elevated BMI accelerates cardiometabolic morbidity. In the same vein, the sign reversal we document for urbanization mirrors Liu et al. (72) observation that early urban growth raises ischemic-heart-disease deaths, while echoing Bandyopadhyay and Green's (73) cross-national evidence that mature urban infrastructures ultimately lower crude mortality rates. This regime-dependent sign change is consistent with Liu et al. (72) and Bandyopadhyay and Green (73), who document that initial urban expansion can raise NCD mortality via lifestyle shifts, while mature urban infrastructure can reverse the trend, and it stands in partial contrast to studies in low-income contexts that found no protective effect even at high urbanization levels. The attenuation of PM2.5's effect in high-happiness regimes aligns with Badida et al.'s (74) meta-analysis showing the strongest pollution hazards in low- and middle-income settings. Finally, our finding that greater per-capita health spending consistently reduces NCD mortality converges with the meta-regression of Gallet and Doucouliagos (75), who reported a robust protective elasticity across 65 studies.

### 4.2 Subjective wellbeing and non-communicable disease mortality rate: panel vector autoregression (panel VAR)

As the panel vector autoregression (panel VAR) framework necessitates the use of stationary variables, we begin by assessing the stationarity properties of our dataset. To this end, we employ the Maddala and Wu (77) panel unit root test, which aggregates the *p*-values from individual unit root tests to evaluate the null hypothesis that all series contain a unit root. When individual intercepts are specified as exogenous variables, the test accommodates heterogeneous mean structures across cross-sectional units. Under the null, the test statistic follows a chi-squared distribution with 238 degrees of freedom. The results, presented in Table 5, indicate that nearly all variables are stationary at the 1% significance level. These findings support the appropriateness of estimating the panel VAR model (Table A3 in Appendix) using the selected variables.

**Table 5:** Panel unit root test.

**Variables**	**Chi square statistic**	***p*-value**
NCD mortality rate (*NCD*_*i, t*_)	2323.5	0.0000
Life ladder (*L*_*i, t*_)	3482.8	0.0000
Alcohol consumption (*D*_*i, t*_)	4271.1	0.0000
Body mass index (*BMI*_*i, t*_)	3397.8	0.0000
Urban population (*N*_*i, t*_)	3227	0.0000
Air pollution (*P*_*i, t*_)	2285.9	0.0000
Control of corruption (*CC*_*i, t*_)	3483.3	0.0000
Health expenditure (*H*_*i, t*_)	3719.1	0.0000
GDP per capita (*Y*_*i, t*_)	3375.8	0.0000

The Maddala and Wu (66) panel unit root test is applied, combining *p*-values from individual ADF tests using Fisher's method. The specification includes individual intercepts as exogenous variables, and the test statistic follows a chi-squared distribution with 238 degrees of freedom.

The reliability of panel VAR model estimation critically depends on the appropriate selection of lag length. Accordingly, it is essential to determine the optimal lag order *p* in the specification. To address this, a set of consistent model and moment selection criteria (MMSC) for GMM estimation is considered. These criteria include the Akaike Information, the Bayesian Information Criterion and the Hannan-Quinn Information Criterion. The results, presented in Table 6, show that a lag length of one minimizes all three criteria. Consequently, the panel VAR estimation is conducted using a lag order of one.

**Table 6:** Optimal lag length.

**Lag**	**AIC**	**BIC**	**HQIC**
1	−1738.182	−6578.884	−3707.41
2	−1721.807	−6453.685	−3654.82
3	−1710.663	−6329.726	−3606.13

Consistent model and moment selection criteria (MMSC) are used to address challenges specific to panel data, including unobserved heterogeneity and cross-sectional dependence.

Following the selection of the optimal lag length, it is essential to assess the stability condition of the panel VAR model, as empirical results are not valid if the system is unstable. Model stability ensures that the impulse response functions converge over time following a shock. To evaluate this, we examine the inverse roots of the lag polynomial's characteristic equation. According to Lutkepohl (62), a panel VAR model is deemed stable if all roots of the characteristic polynomial lie within the unit circle i.e., if their moduli are strictly less than one. As illustrated in Figure A2 in Appendix, all roots of the companion matrix are located inside the unit circle, confirming that the estimated panel VAR model satisfies the stability condition.

With the stability condition verified and the optimal lag length established, we proceed to estimate the panel VAR model. The specification assumes the weak exogeneity of *Control of Corruption*, in line with existing literature that treats institutional quality indicators as exogenous determinants of health outcomes (78). All other variables, which are expected to exhibit dynamic interdependence, are treated as endogenous within the system.

Granger causality test evaluates whether the inclusion of lagged values of a predictor variable improves the forecast of a dependent variable, compared to a model with only the lagged dependent variable. This is implemented through separate Wald tests under the null hypothesis that the excluded variable does not Granger cause the dependent variable, against the alternative that it does (79). At the 5% significance level, the null hypothesis is rejected when the associated *p*-value is < 0.05.

The results, presented in Table 7, indicate that the Life Ladder score Granger causes NCD mortality rate, as the null hypothesis is rejected at the 5% level. Further, the null hypothesis that NCD mortality rate does not Granger cause the Life Ladder score is also rejected, suggesting a bidirectional relationship between these two variables. Similarly, bidirectional Granger causality is observed between NCD mortality rate and both obesity prevalence and urbanization rate, implying mutual predictive relationships (79).

**Table 7:** Granger causality results for NCD mortality rate.

**Null hypothesis**	***F*-statistic**	**Prob**.
Life ladder does not Granger cause NCD mortality rate	4.24	0.0396
NCD mortality rate does not Granger cause life ladder	3.06	0.0807
Alcohol consumption does not Granger cause NCD mortality rate	3.02	0.0825
NCD mortality rate does not Granger cause alcohol consumption	0.07	0.7882
Obesity prevalence does not Granger cause NCD mortality rate	15.74	0.0001
NCD mortality rate does not Granger cause obesity prevalence	35.87	0.0001
Urban population does not Granger cause NCD mortality rate	4.71	0.0301
NCD mortality rate does not Granger cause urban population	5.58	0.0183
Air pollution does not Granger cause NCD mortality rate	3.04	0.0814
NCD mortality rate does not Granger cause Air pollution	0.79	0.3750
Health expenditure does not Granger cause NCD mortality rate	0.01	0.9433
NCD mortality rate does not Granger cause Health expenditure	1.78	0.1824
GDP per capita does not Granger cause NCD mortality rate	0.00	0.9982
NCD mortality rate does not Granger cause GDP per capita	0.00	0.9990

However, air pollution Granger causes NCD mortality rate in a unidirectional manner, while health expenditure and GDP per capita do not exhibit statistically significant Granger causality with respect to NCD mortality rate. These findings underscore the relevance of subjective wellbeing, behavioral, demographic, and environmental factors as key predictors of NCD mortality across the sample of 123 countries (36).

Finally, by examining the estimated coefficients presented in Table 6, we find that behavioral and demographic variables such as alcohol consumption, obesity prevalence (BMI), urbanization rate, and air pollution exhibit similar directional effects across both the PSTR and panel VAR models, thereby reinforcing the robustness of these associations. However, unlike the PSTR results, where certain economic variables (e.g., health expenditure and GDP per capita) were found to be significant under regime-specific conditions, the panel VAR estimation reveals that the lagged values of these economic indicators do not significantly predict current mortality outcomes.

This divergence in results does not undermine the earlier findings; rather, it suggests that the effects of economic variables may be non-linear or dependent on underlying regimes, features more effectively captured within the PSTR framework. In contrast, the panel VAR model, which assumes linear dynamics and homogeneity in lagged effects, may not fully reflect such conditional relationships. Nevertheless, the panel VAR results underscore important dynamic interdependencies among key variables, particularly in the case of mortality rate and obesity prevalence, offering valuable insights into the temporal structure of these relationships.

**Figure 2:**
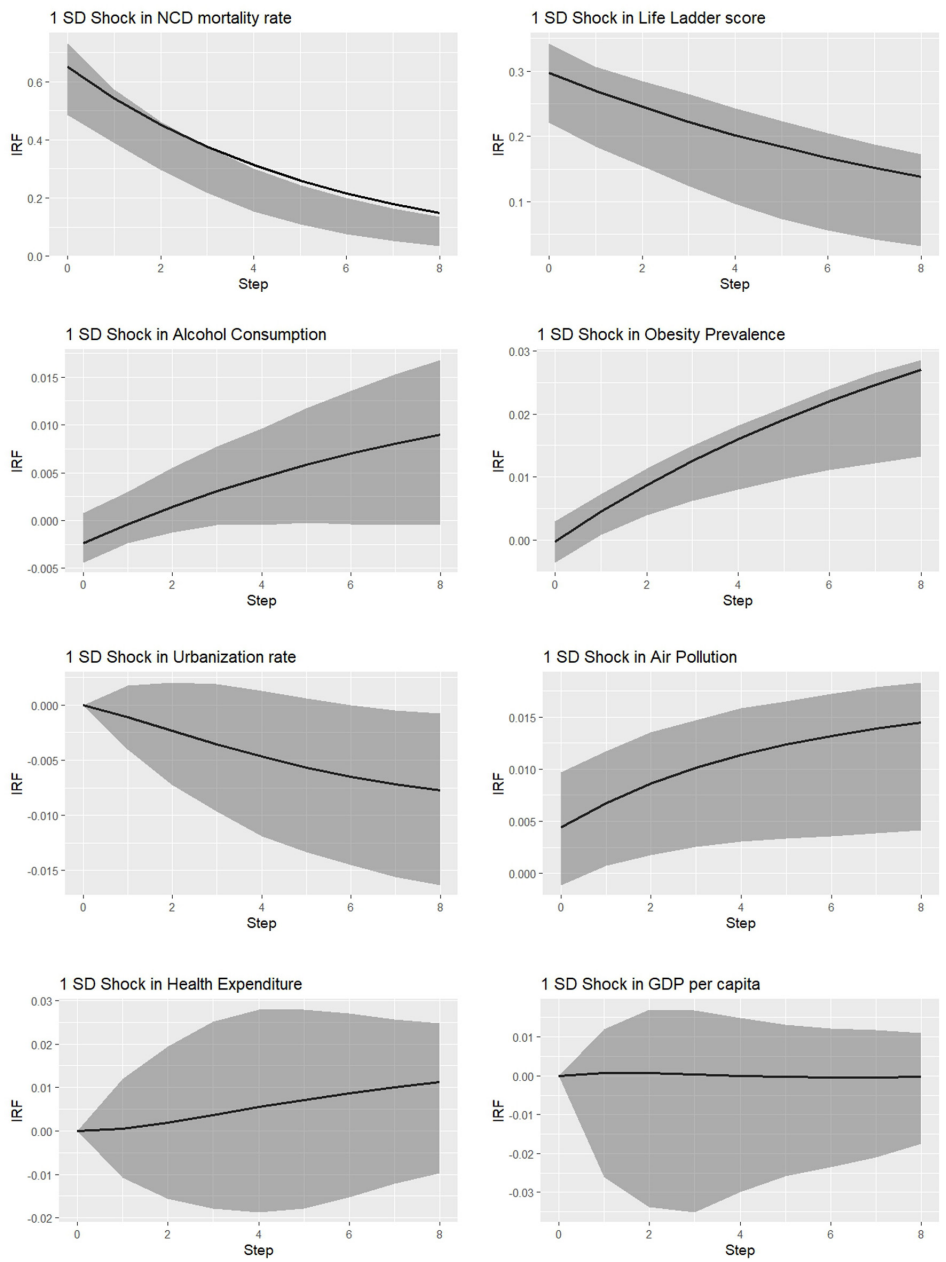
Responses of NCD mortality rate.

To gain deeper insights into the dynamic relationships between the explanatory variables and NCD mortality rates, we extend the analysis by estimating impulse response functions (IRFs). An IRF examines the response of a dependent variable following a one-time shock to an endogenous variable, holding all other innovations constant. Figure 2 presents the IRFs of NCD mortality rate resulting from one-unit shocks to each of the endogenous variables.

The inverse dynamic response of NCD mortality to its own shock suggests a tendency toward mean reversion, indicating that the system gradually corrects deviations from its equilibrium path. Furthermore, a positive shock to the Life Ladder score yields a negative and significant response in NCD mortality rates, highlighting the mitigating influence of subjective wellbeing (44). In contrast, positive shocks to alcohol consumption, obesity prevalence (BMI), and air pollution result in upward responses in NCD mortality, consistent with the well-documented epidemiological risks (80). Interestingly, a positive shock to urbanization is associated with a reduction in NCD mortality, possibly reflecting improved access to health infrastructure or services in urban areas (81).

In the case of health expenditure and GDP per capita, the impulse responses of NCD mortality rates remain statistically insignificant, as illustrated by the 95% confidence intervals (shaded regions in the graphs), indicating weak dynamic interdependence of these economic variables with NCD mortality in the panel VAR context.

## 5 Discussion

Across the 123-country panel analyzed from 2006 to 2021, the Panel-Smooth-Transition-Regression identifies a single statistically relevant threshold on the Cantril Life-Ladder at roughly 2.7 points. Once a nation's average happiness rises above this value, each one-per-cent increase in subjective wellbeing is linked to an estimated 0.429% fall in the 30-to-70-year non-communicable-disease mortality rate, even after controlling for behavioral, demographic, environmental, economic and institutional factors, whereas below that threshold the coefficient is small and not statistically different from zero, indicating that marginal improvements in happiness at very low levels do not yet translate into measurable reductions in NCD deaths. Within the observed empirical range (from 2.18 to 7.97 ladder points) the regression surface slopes steadily downward, and neither the PSTR nor the panel-VAR impulse-response functions reveal any point at which the health effect of additional happiness becomes insignificant or turns harmful; on the contrary, a positive happiness shock continues to produce a durable, statistically significant decline in NCD mortality for several subsequent years with no sign of reversal. Consequently, the data show that the protective influence of subjective wellbeing begins only after the population has moved beyond the lower-wellbeing regime but, once initiated, remains beneficial throughout the spectrum, providing no evidence of a “too-much-happiness” penalty.

Compared with prior research that assumes a linear association between happiness and health outcomes [e.g., (17, 18)], our findings demonstrate a distinct non-linear pattern with a critical threshold at ~2.7 Life-Ladder points. This threshold indicates that the protective effect of happiness on NCD mortality emerges only once populations exceed a minimal baseline of subjective wellbeing, a nuance absents from earlier analyses. While some studies have hinted at context-dependent or diminishing returns to happiness [e.g., (82, 83)], none, to our knowledge, have empirically identified such a tipping point using a large cross-country panel and a Panel Smooth Transition Regression framework. This divergence underscores our study's novel contribution in quantifying the threshold and linking it to policy-relevant socio-economic and institutional correlates.

The 2.7 life-ladder threshold suggests health gains will remain limited until governments raise wellbeing above roughly the third rung on the Cantril scale, with model covariates showing how to achieve and sustain it. This 2.7-point Life-Ladder threshold may reflect the minimum socio-economic and institutional conditions required for happiness to translate into measurable health gains. Countries clustered just above this value tend to exhibit higher per-capita health expenditure, lower corruption, stronger social safety nets, and more stable governance relative to those below the threshold, suggesting that these enabling contexts may amplify the protective effect of subjective wellbeing on NCD mortality. Because obesity prevalence and alcohol consumption retain strong positive associations with premature NCD mortality on both sides of the threshold, policy strategies that foster happiness by encouraging healthy diets, limiting marketing of calorie-dense foods, tightening alcohol taxation and availability, and embedding active-living infrastructure in city design are likely to produce a dual dividend: they push average wellbeing upward while simultaneously weakening two of the most powerful behavioral conduits to chronic disease. In countries that still fall below the threshold, the insignificant happiness coefficient underscores that these behavioral levers need to be complemented by broader structural reforms (most notably the expansion of per-capita health expenditure and the improvement of institutional integrity) because the analysis shows that greater spending on care and tighter control of corruption each exert an independent downward pull on NCD mortality yet also correlate positively with life-evaluation scores.

Urbanization and air quality represent a second layer complication of policy nuance. The data indicate that while rapid urban development is initially associated with more NCD mortality—it becomes protective once a country enters the higher wellbeing regime. This suggests that urban development should be managed carefully so that the social and economic prospects that increase happiness are not compromised by pollution and sedentary quality of life. Thus, investments in clean public transport, green space, and complex particulate-matter reduction, serve the dual aims of enhancing subjective wellbeing and disrupting the environmental pathway toward chronic disease. Furthermore, as the health return to happiness continues to strengthen rather than diminish within the observed range of happy (to eight life-ladder points) the findings justify continual investment in psychosocial quality of life, even in higher-income contexts where GDP per capita is already yielding a direct protective benefit: policies that reduce inequality, increase social trust and build community connection, or support mental-health service provision can still convert incremental increases in happiness into epidemiologically measurable reductions in premature mortality.

Finally, the discovery of bidirectional causality between wellbeing and NCD mortality suggests that functional prevention automatically creates a virtuous cycle; as deaths decline, life satisfaction rises further, thereby maximizing the effectiveness of all future health and social investments. This bidirectional association between happiness and NCD mortality may be sustained by behavioral feedback loops, whereby greater happiness promotes healthier lifestyles (such as increased physical activity and reduced harmful consumption) which in turn reduce NCD risk and reinforce subjective wellbeing, a dynamic consistent with prior evidence on reciprocal links between wellbeing and health behaviors (8, 11, 66). Consequently, policy-makers should consider subjective wellbeing both a target and an intermediary: raising wellbeing beyond the 2.7-point threshold activates the protective agent identified by the Panel-Smooth-Transition-Regression, whilst at the same time, the accumulative nature of areas of behavioral, demographic, environmental and institutional risk continues to compound the health benefit of each step ascended on the life ladder.

## 6 Implications

### 6.1 Theoretical contributions

We theorize happiness as a threshold-dependent population health asset that complements structural determinants once a minimum level of subjective wellbeing is met. Leveraging our PSTR results, we formalize a tipping point at roughly 2.7 Life-Ladder points, above which each 1% increase in happiness reduces 30–70 NCD mortality by about 0.43%, while below it the marginal effect is negligible—clarifying why prior linear models obscured this non-linearity. We further contribute a regime-dependence account: the signs/magnitudes of covariates shift across happiness regimes—urbanization turns from harmful to protective, PM_2.5_ attenuates to insignificance, and GDP per capita becomes protective only at higher happiness—implying complementarity between psychosocial and infrastructural conditions. By contrast, health expenditure is consistently protective in both regimes, indicating a baseline lever that operates independent of the threshold. We also demonstrate bidirectional dynamics between happiness and NCD mortality (PVAR), providing a mechanistic foundation for virtuous cycles in which mortality declines and wellbeing reinforce each other over time. Finally, we integrate these findings into a conceptual model in which happiness functions as a capacity multiplier only after core socio-economic and institutional conditions are present, reconciling mixed results in earlier linear studies and specifying when (and why) wellbeing translates into population survival benefits.

### 6.2 Managerial implications

At the national level, the threshold finding should inform regime-based segmentation and KPI design. Ministries of health, public insurers, and national observatories should track the country's Life-Ladder mean and the population share below the threshold; in countries with Life-Ladder ≤ 2.7, foundational enablers—accessible primary and mental health care, navigation support, and financial protection—should be prioritized, as incremental happiness gains are unlikely to reduce mortality without structural improvements. National authorities should fund proven programs targeting obesity (nutrition counseling, activity pathways) and harmful alcohol use (SBIRT, relapse prevention), as both remain robust drivers of NCD mortality across regimes. In low-happiness countries, authorities should mitigate PM_2.5_ through stricter standards, enforcement, and high-risk clinical protocols. In higher-happiness settings, policymakers should prioritize urban-health design (active-transport infrastructure, green-space and park-prescription initiatives, and transit enhancements) to leverage urbanization's protective effect. Public budgets should protect a ring-fenced prevention share, consistent with the protective effect of health expenditure in both regimes, and national dashboards should trigger corrective actions when Life-Ladder or risk indicators drift. Program evaluation should be institutionalized (A/B rollouts, stepped-wedge trials, and impulse-response–style follow-ups) to detect the expected virtuous cycle (happiness improvements followed by sustained mortality declines). Resources should be reallocated toward interventions demonstrating regime-consistent returns.

### 6.3 Policy implications

At the national level, governments should aim to lift populations above the ≈2.7 Life-Ladder threshold while concurrently addressing the principal behavioral and environmental conduits of NCD mortality. In practice, alcohol availability and marketing ought to be tightened, with fiscal instruments (e.g., excise taxes, minimum unit pricing) deployed to curb harmful use. Population-wide obesity prevention should be scaled through nutrition standards, front-of-pack labeling, and active-living infrastructure, given the persistence of these risks across regimes. Investment in clean air is warranted (stringent PM_2.5_ standards and robust enforcement) with priority to low-happiness regions where the mortality link is strongest. Urbanization policies should be steered toward walkability, mass transit, and accessible green space to realize the protective effects observed in higher-happiness contexts. Per-capita health expenditure should expand with a ring-fenced share for prevention and chronic care, a lever operating in both regimes. Inclusive growth strategies are needed to convert income gains into health only once psychosocial and system prerequisites are in place, recognizing that GDP appears protective mainly in higher-happiness settings. Finally, national wellbeing policies should be aligned with health targets and monitoring, acknowledging bidirectional causality: raising happiness above the threshold initiates mortality declines that subsequently reinforce wellbeing and magnify medium-term policy returns.

## 7 Conclusion

The evidence collected across 123 countries from 2006 to 2021 shows that subjective wellbeing operates as a genuine, quantifiable determinant of non-communicable-disease mortality, yet its protective effect materializes only once national life-ladder scores rise beyond a threshold of roughly 2.7 points. Above that level, incremental gains in happiness translate into progressively larger percentage reductions in premature NCD deaths, and neither the panel-smooth-transition estimates nor the panel-VAR impulse responses indicate any point at which the marginal benefit becomes statistically negligible or reverses direction. That finding not only refines the Easterlin-type intuition that “more happiness is better” but also qualifies it: very low wellbeing must first be remedied before the health dividend is unlocked, after which the dividend appears open-ended within the observed range. In addition to this major finding, the analysis establishes that behavioral (obesity, alcohol consumption), demographic (urbanization), environmental (fine-particulate pollution), and economic-institutional (health expenditure, income, governance quality) variables have independent, somewhat regime-specific associations with mortality, reinforcing the point that policies seeking to enhance happiness cannot neglect the structural determinants that together shape both disease risk and life evaluation.

With respect to the two primary research questions, our results confirm that national Life-Ladder scores must climb above an empirically estimated 2.7-point threshold before happiness delivers a measurable survival benefit, each additional 1% increase thereafter cutting 30-to-70-year NCD mortality by roughly 0.43%. Across the entire upper range of observed scores (2.7–7.97), this protective elasticity remains negative and statistically robust, indicating no point at which further gains in happiness become neutral or harmful. Turning to the broader question of whether subjective wellbeing exerts complex, non-linear effects on health, our Panel Smooth Transition Regression (PSTR) model, corroborated by impulse-response analysis, delineates a two-regime structure in which only populations whose Life-Ladder scores exceed the 2.7-point threshold experience a sustained decline in NCD mortality after a positive happiness shock.

Limitations include the vulnerability of self-reported Life-Ladder scores to measurement error, cross-cultural response styles, and reporting bias; possible selection bias from under-coverage of low-income or conflict-affected settings in the underlying surveys and health statistics [see, e.g., (84)]; the inability of country-level aggregates to capture subnational heterogeneity in happiness and health; and residual endogeneity (reverse causality and omitted variables) despite controls and PVAR tests—future work should integrate subnational micro-data and multilevel designs, expand coverage to fragile states, and employ stronger identification (e.g., IV/GMM or IV-PSTR) while triangulating survey wellbeing with alternative indicators. A major limitation is measurement error impacting the Life-Ladder scores and WHO mortality statistic and Gallup's coverage potentially overlooks many low-income or conflict states that may lead to panel bias. National aggregated data hides heterogeneity within countries by age, sex, income and geographic region, and the Life-Ladder data analysis being confined to ages 30–70 omits morbidity, disability, and other mortality measures from longevity in future studies.

Future work would include metrics on years lived with disability or impairment, metabolic biomarkers, hospital admission records, etc., to hypothesize whether greater levels of happiness delay disease onset or its severity or compress mortality by sheer happenstance, as well as explore how these relationships vary across the socio-economic strata of any given country.

## Data Availability

The raw data supporting the conclusions of this article will be made available by the authors, without undue reservation.
