# Supplementary material for: How happy is healthy enough? Uncovering the happiness threshold for global non-communicable disease prevention
Source: Front Med (Lausanne). 2025 Oct 21;12:1667645. doi: 10.3389/fmed.2025.1667645 (PMC12583178; doi:10.3389/fmed.2025.1667645)

Appendix

Table 1A: Evaluation results of two-regime PSTR model

Transition variable: Life Ladder ($L_{i,(t-1)}$)	
No remaining heterogeneity (*r*=*1*)	0.8585(0.7325)
Parameter constancy (*r*=*1*)	0.3088 (1.000)

Note: The table reports the HAC F-test statistic for no remaining nonlinearity and parameter constancy. P-values are reported in parentheses. The PSTR models non-communicable disease mortality rates using lagged life ladder, alcohol consumption, urban population, air pollution, control of corruption, health expenditure, and GDP per capita as an explanatory variables. The analysis uses a balanced panel of 123 countries from 2006 to 2021.

Table 2A: Transition function statistics

Year	Perc. of Countries with $g\left(L_{i,(t-1)}, \gamma, c\right) > 0.5$	Value of $g\left(L_{i,(t-1)}, \gamma, c\right) > 0.5$		
		Median	25%	75%
2007	48	0.615	0.560	0.686
2008	45.5	0.624	0.560	0.692
2009	46.3	0.634	0.556	0.688
2010	51.2	0.615	0.543	0.681
2011	49.6	0.621	0.562	0.691
2012	47.2	0.638	0.552	0.688
2013	47.2	0.621	0.565	0.685
2014	46.3	0.635	0.573	0.691
2015	48.8	0.628	0.570	0.677
2016	49.6	0.618	0.563	0.665
2017	52	0.598	0.567	0.668
2018	53.7	0.611	0.571	0.682
2019	56.1	0.607	0.568	0.670
2020	58.5	0.609	0.579	0.674
2021	57.7	0.605	0.565	0.660

Note: The table presents summary statistics for value of transition function in PSTR model for NCD mortality rates with lagged life ladder, alcohol consumption, urban population, air pollution, control of corruption, health expenditure, and GDP per capita as regressors and lagged life ladder as transition variable. The analysis uses a balanced panel of 123 countries from 2006 to 2021.

Table 3A: Results from panel VAR model

	$NCD_{i,t}$	$L_{i,t}$	$D_{i,t}$	$BMI_{i,t}$	$N_{i,t}$	$P_{i,t}$	$H_{i,t}$	$Y_{i,t}$
$L. NCD_{i,t}$	0.8326***	-10.1158*	0.0014	0.0077***	0.0040**	0.0486	-4.4698	0.0529
	(0.0115)	(5.7873)	(0.0053)	(0.0013)	(0.0017)	(0.0548)	(3.3510)	(43.8921)
$L. L_{i,t}$	-0.0001**	0.6385***	0.00002	9.75e-06**	5.55e-06	-0.0003	0.0350***	0.3450**
	(0.00004)	(0.0199)	(0.00001)	(4.44e-06)	(5.89e-06)	(0.0002)	(0.0115)	(0.1512)
$L.D_{i,t}$	0.0549**	-2.6200	0.9054***	0.0045	0.0094**	0.1443	3.1109	-54.1878
	(0.0246)	(12.379)	(0.0112)	(0.0028)	(0.0037)	(0.1173)	(7.1684)	(93.8920)
$L. BMI_{i,t}$	0.0451***	4.7548	-0.0097*	1.0200***	0.0103***	-0.2232***	6.9411**	99.4977**
	(0.0114)	(5.7222)	(0.0052)	(0.0013)	(0.0017)	(0.0542)	(3.3134)	(43.3983)
$L. N_{i,t}$	-0.0225**	-7.9586	-0.0152***	-0.0008	0.9774***	-0.0015	-2.4780	-30.4121
	(0.0104)	(5.2136)	(0.0047)	(0.0012)	(0.0015)	(0.0494)	(3.0189)	(39.5410)
$L. P_{i,t}$	0.0073*	1.5302	0.0049**	0.0008	-0.0014**	0.6058***	-1.6143	-5.0313
	(0.0042)	(2.1007)	(0.0019)	(0.0005)	(0.0006)	(0.0199)	(1.2164)	(15.9324)
$L. H_{i,t}$	4.77e-06	-0.0577*	-0.00003	-0.00007***	-3.40e-06	-0.0001	0.8803***	-0.0255
	(0.00007)	(0.0337)	(0.00003)	(7.51e-06)	(9.97e-06)	(0.0003)	(0.0195)	(0.2559)
$L. Y_{i,t}$	1.46e-08	0.0034	-5.15e-06*	3.45e-06***	-1.10e-07	0.00002	-0.0067***	0.6997***
	(6.59e-06)	(0.0033)	(3.01e-06)	(7.38e-07)	(9.79e-07)	(0.00003)	(0.0019)	(0.0251)

Note: Standard errors are reported in parentheses. *** $p < 0.01$, ** $p < 0.05$, * $p < 0.10$.

Table 4A: Sensitivity analysis of PSTR estimation

	**Low Healthy Life expectancy countries**	**High Healthy Life expectancy countries**
Dependent Variable: NCD mortality rate ($NCD_{i,t}$)		
Explanatory Variables:		
L.NCD mortality rate ($NCD_{i,t-1}$)	0.537***(0.112)	1.025***(0.101)
Health Life Expectancy ($L_{i,(t-1)}$)	-0.0304(0.0358)	-0.9604** (0.518)
Alcohol Consumption ($D_{i,(t-1)}$)	0.06*** (0.011)	0.04***(0.006)
Body Mass Index ($BMI_{i,(t-1)}$)	0.0158*** (0.0064)	0.0281**(0.007)
Urban population ($N_{i,(t-1)}$)	0.336*** (0.141)	-0.313***(0.179)
Air pollution ($P_{i,(t-1)}$)	0.054*** (0.058)	0.066(0.072)
Control of Corruption ($CC_{i,(t-1)}$)	-0.046(0.038)	-0.028(0.076)
Health expenditure ($H_{i,(t-1)}$)	-0.030**(0.004)	-0.021**(0.006)
GDP per capita ($Y_{i,(t-1)}$)	0.003(0.039)	-0.012***(0.007)
Location parameter (c)	4.132(0.067)	
Slope parameter (γ)	2.717 (0.727)	
Notes: The standard errors for coefficients in parentheses are corrected for heteroskedasticity. *** p < 0.01, ** p < 0.05, * p < 0.10.		

Figure 1A: Estimated transition function of the PSTR model

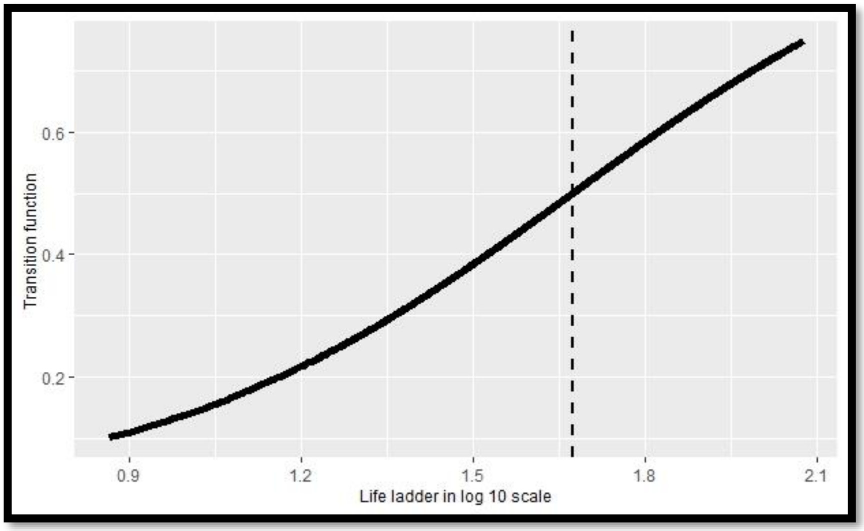

Figure 2A: Stability of panel VAR Model

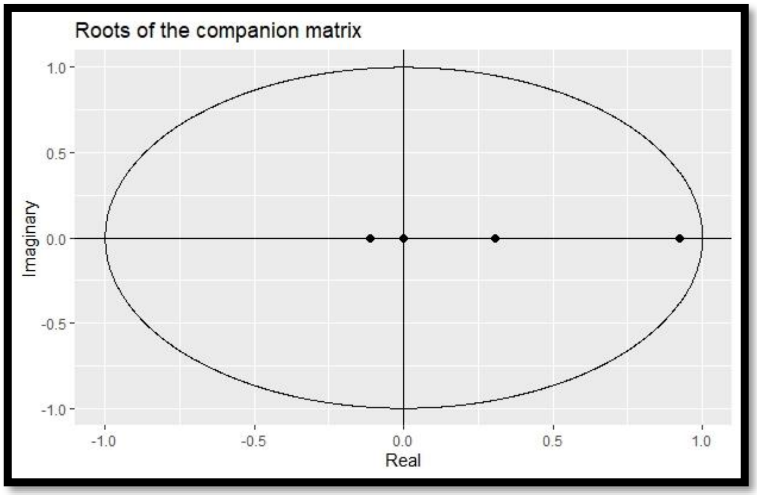

Supplement: Supplementary file 1 [file Table_1.pdf]
